# Cyclic RGD and *iso*DGR Integrin Ligands Containing *cis*-2-amino-1-cyclopentanecarboxylic (*cis*-β-ACPC) Scaffolds

**DOI:** 10.3390/molecules25245966

**Published:** 2020-12-16

**Authors:** Silvia Panzeri, Daniela Arosio, Silvia Gazzola, Laura Belvisi, Monica Civera, Donatella Potenza, Francesca Vasile, Isabell Kemker, Thomas Ertl, Norbert Sewald, Oliver Reiser, Umberto Piarulli

**Affiliations:** 1 Dipartimento di Scienza e Alta Tecnologia, Università degli Studi dell’Insubria, Via Valleggio 11, 22100 Como, Italy; silvia.panzeri.87@gmail.com (S.P.); s.gazzola@uninsubria.it (S.G.); 2 Institute of Organic Chemistry, University of Regensburg, Universitätsstraße 31, 93053 Regensburg, Germany; thomas.ertl@chemie.uni-regensburg.de (T.E.); Oliver.Reiser@chemie.uni-regensburg.de (O.R.); 3 Consiglio Nazionale delle Ricerche (CNR), Istituto di Scienze e Tecnologie Chimiche (SCITEC), Giulio Natta, Via C. Golgi 19, 20133 Milan, Italy; daniela.arosio@scitec.cnr.it; 4 Dipartimento di Chimica, Università degli Studi di Milano, Via C. Golgi 19, 20133 Milan, Italy; laura.belvisi@unimi.it (L.B.); monica.civera@unimi.it (M.C.); donatella.potenza@unimi.it (D.P.); francesca.vasile@unimi.it (F.V.); 5 Department of Chemistry, Organic and Bioorganic Chemistry, University of Bielefeld, Universitätsstraße 25, 33615 Bielefeld, Germany; isabell.kemker@uni-bielefeld.de (I.K.); norbert.sewald@uni-bielefeld.de (N.S.)

**Keywords:** peptidomimetics, integrin ligands, beta-amino acids, NMR conformational analysis

## Abstract

Integrin ligands containing the tripeptide sequences Arg-Gly-Asp (RGD) and *iso*-Asp-Gly- Arg (*iso*DGR) were actively investigated as inhibitors of tumor angiogenesis and directing unit in tumor-targeting drug conjugates. Reported herein is the synthesis, of two RGD and one *iso*DGR cyclic peptidomimetics containing (1*S*,2*R*) and (1*R*,2*S*) *cis*-2-amino-1-cyclopentanecarboxylic acid (*cis*-β-ACPC), using a mixed solid phase/solution phase synthetic protocol. The three ligands were examined in vitro in competitive binding assays to the purified α_v_β_3_ and α_5_β_1_ receptors using biotinylated vitronectin (α_v_β_3_) and fibronectin (α_5_β_1_) as natural displaced ligands. The IC50 values of the ligands ranged from nanomolar (the two RGD ligands) to micromolar (the isoDGR ligand) with a pronounced selectivity for α_v_β_3_ over α_5_β_1_. In vitro cell adhesion assays were also performed using the human skin melanoma cell line WM115 (rich in integrin α_v_β_3_). The two RGD ligands showed IC_50_ values in the same micromolar range as the reference compound (*cyclo*[RGDfV]), while for the *iso*DGR derivative an IC_50_ value could not be measured for the cell adhesion assay. A conformational analysis of the free RGD and *iso*DGR ligands by NMR (VT-NMR and NOESY experiments) and computational studies (MC/EM and MD), followed by docking simulations performed in the α_V_β_3_ integrin active site, provided a rationale for the behavior of these ligands toward the receptor.

## 1. Introduction

Integrins are a large family of heterodimeric cell adhesion protein receptors involved in physiological and pathological processes concerning cell adhesion, cell motility, and cell survival [1,2]. In particular, α_v_β_3_, α_v_β_5_, and α_5_β_1_ integrins are involved in tumor angiogenesis and are overexpressed in tumor vascular tissues [3,4,5]. These integrins recognize and bind the Arg-Gly-Asp (RGD) sequence in their natural ligands [6], but also the *iso*DGR sequence was shown to fit into the RGD-binding pocket of α_v_β_3_ integrin, establishing the same interactions [7,8,9,10]. For both sequences, flanking residues combined to the 3D presentation determine the recognition specificity. Since the pioneering work of Kessler and coworkers [11], many different RGD peptides and peptidomimetics have been developed as integrin ligands and investigated as potential antitumor drugs with antiangiogenic properties [12,13,14] or directing ligands in molecular imaging and targeted anticancer therapy, which emerged as powerful weapon for reducing the toxicity of the antitumor treatments and the insurgence of drug resistance [15,16,17,18,19,20,21].

**Figure 1:**
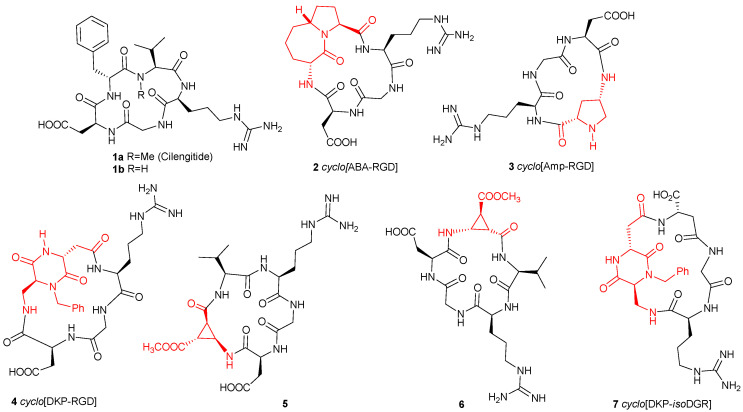
Cyclic RGD and *iso*DGR integrin ligands (azabicyclic alkane (ABA), aminoproline (Amp), and diketopiperazine scaffolds (DKP)).

In many cases, the RGD sequence is constrained in cyclic peptides such as the cyclopentapeptide *cyclo*[Arg-Gly-Asp-*N*Me Val-D-Phe], Cilengitide **1a** [22], the most studied ligand and forefather of a whole series of cyclic ligands, in which the complementary dipeptide sequence was varied to optimize the interaction of the RGD sequence with the integrin receptor. Based on this notion, several peptidomimetic scaffolds were inserted to mimic this complementary dipeptide moiety such as the bicyclic lactam in **2** [23,24,25], the 4-aminoproline in **3** [26], or the bifunctional diketopiperazine in **4 [27,28]** (Figure 1).

By contrast, the number of *iso*DGR containing cyclic peptides and peptidomimetics which were synthesized and tested is much more limited and, among them, the cyclic *iso*DGR compound **7** (Figure 1) containing the bifunctional diketopiperazine mentioned above is one of the few low nM α_v_β_3_ binder reported so far [29,30,31,32].

β-Amino acids have received considerable attention as possible substituents of α-amino acids in peptides to probe the structural specificity of α-amino acid-binding sites or as inhibitors of enzymes [33]. In addition, their incorporation into peptides of pharmacological interest was sometimes advantageous in terms of biological activity, metabolic stability, and conformational characteristics. In particular, β-amino acids stabilize distinct overall conformations of cyclopeptides and they act as γ-turn mimetics: [34,35], if a single β-amino acid is incorporated into a cyclic pentapeptide, it preferably occupies the central position of a γ-turn. These conformational preferences were thoroughly investigated in the field of RGD-cyclopeptides by the introduction of β-aminocyclopropane carboxylic acids (β-ACCs) [36], which are among the most restricted β-alanine derivatives, and whose rigidity is conferred by the small-sized ring closure. Reiser and co-workers synthesized both *cis*- and *trans*-β-aminocyclopropane carboxylic acids [37] and incorporated two enantiomeric *cis*-β-aminocyclopropanecarboxylic acids (*cis*-β-ACC) into two 16-membered cyclic RGD peptidomimetics **5**–**6** (Figure 1). These peptides showed nanomolar affinity toward α_v_β_3_ and α_5_β_1_ integrins in in vitro cell adhesion assays.

**Figure 2:**
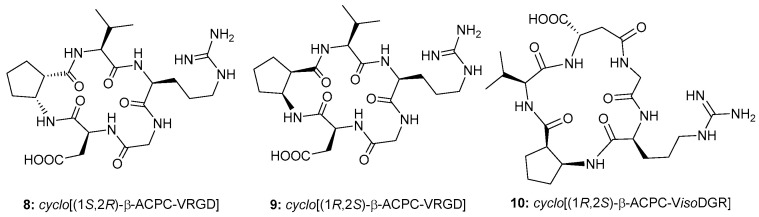
Cyclic peptidomimetics containing *cis*-β-ACPC scaffolds.

These considerations prompted us to investigate the properties of other β-aminocycloalkane carboxylic acids and herein we describe the synthesis, the conformational analysis, and some biological investigation of two RGDs, **8**–**9** (Figure 2), and one *iso*DGR, **10** (Figure 2) cyclic peptidomimetics, containing two *cis*-2-amino-1-cyclopentanecarboxylic (*cis*-β-ACPC) scaffolds. The RGD ligands feature a 16-membered ring containing the RGD sequence as well as a Val residue and either (1*S*,2*R*)-*cis*-β-ACPC (**8**) or (1*R*,2*S*)-*cis*-β-ACPC (**9**). In the case of the *iso*DGR ligand only the (1*R*,2*S*)-*cis*-β-ACPC scaffold was used and a 17-membered ring is obtained, since the Asp residue participates to the sequence through its β-carboxylic group (*iso*Asp residue). Integrin receptor competitive binding assays (Table 1) and cell adhesion assays with an α_V_β_3_ positive cell line (WM115, a human skin melanoma cell line) (Table 2) were performed for all the compounds. The conformational preferences of the free ligands were investigated by NMR and computational methods. The conformational study of cyclic peptides and peptidomimetics is generally a complex matter, yet an essential step prior to docking calculations that are in most cases unable to perform a rigorous sampling of the macrocycle conformations [38,39]. In this paper, minimized structures satisfying the characteristic NOE contacts and H-bonds were employed as starting geometries for docking studies in the α_v_β_3_ integrin.

## 2. Results

2-Aminocyclopentanecarboxylic acids (β-ACPCs) and their heterocyclic analogs represent a widely studied class of cyclic unnatural β-amino acids, showing interesting biological and conformational properties. For example, *cis*-(1*R*,2*S*)-2-aminocyclopentanecarboxylic acid (Cispentacin) is a potent antifungal agent [40], racemic *cis*-4-aminopyrrolidine-3-carboxylic acid has been used to probe the structure of the GABA (gamma-aminobutyric acid) receptor [41], while *cis*-*N*-Boc-4-aminopyrrolidine-3-carboxylic acid is a modestly active influenza neuraminidase inhibitor [42]. 2-Aminocyclopentanecarboxylic acid and 4-aminopyrrolidine-3-carboxylic acid have also found applications in the area of foldamers as promoters of helical structures, both as oligomeric structures or in combination with α-amino acids [43,44], and were recently used by Reiser and co-workers for the synthesis of helical peptidic foldamers in the synthesis of Neuropeptide Y analogs [45,46].

### 2.1. Synthesis

**Scheme 1:**
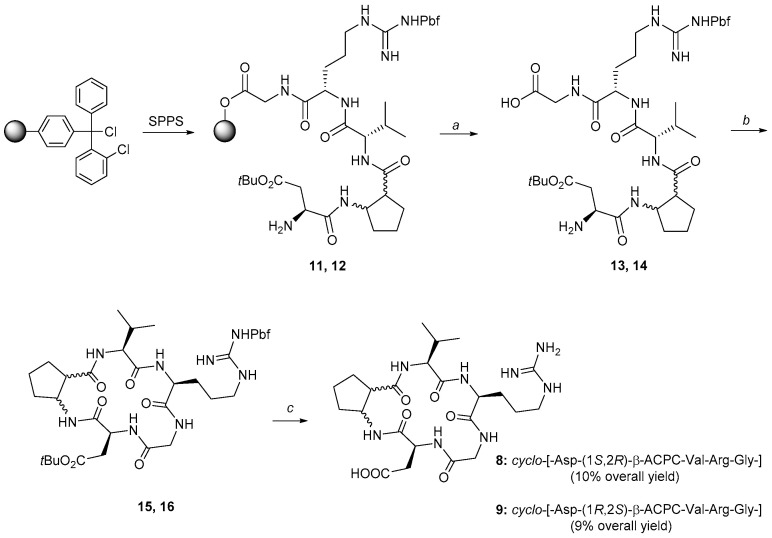
Synthesis of *cyclo*-[-Arg-Gly-Asp-(1*S*,2*R*)-β-ACPC-Val-] (**8**) and *cyclo*-[-Arg-Gly-Asp-(1*R,*2*S*)-β-ACPC-Val-] (**9**) via solid phase peptide synthesis of the linear counterparts **13** and **14** after cleavage from the resin, via in solution phase macrocyclization (**15** and **16**) and their full deprotection. a: 1% TFA in DCM, rt, 5 min for each cycle; b: HATU, DIPEA, DMF, rt, 30 min. c: TFA/Et_3_SiH/H_2_O, 95%/2.5%/2.5%, rt, 8 h. DIPEA = di*iso*propyl ethylamine, TFA = trifluoroacetic acid.

For the synthesis of the cyclic peptidomimetics **8**–**9** either (1*S*,2*R*)-*cis*-β-ACPC or (1*R*,2*S*)-*cis*-β-ACPC were used. The synthesis of the cyclic peptidomimetics was obtained by a mixed solid phase/solution phase approach (Scheme 1). The linear precursors **11** and **12** were assembled by SPPS on *O*-chlorotritylchloride resin as solid support using a Fmoc protection on the α-amino groups. Fmoc-ortogonal protecting groups were selected for the side chains of the amino acids (Pbf for Arg and *t*Bu for Asp). The cleavage of the linear precursor from the resin was accomplished in weakly acidic conditions.

The macrocyclization on compounds **13** and **14** was performed under pseudo-high dilution conditions [47], and the final deprotection was carried out by treatment with TFA/Et_3_SiH/water, 95%/2.5%/2.5% and was followed by purification of the cyclic peptidomimetics by preparative HPLC. An overall yield of 10% and 9% and a purity of 96% and 99% was obtained for compounds **8** and **9**, respectively.

**Scheme 2:**
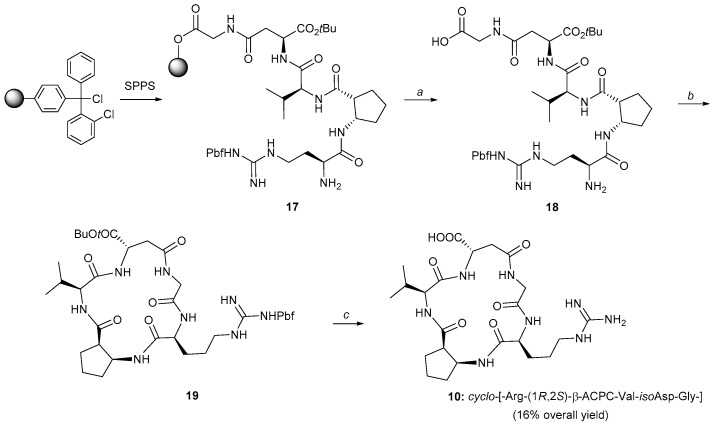
Synthesis of *cyclo*-[-*iso*Asp-Gly-Arg-(1*R,*2*S*)-β-ACPC-Val-] (**10**) via solid phase peptide synthesis of the linear counterpart **18** after cleavage from the resin, via in solution phase macrocyclization (**19**) and its full deprotection. a: 1% TFA in DCM, rt, 5 min for each cycle; b: HATU, DIPEA, DMF, rt, 30 min. c: TFA/Et_3_SiH/H_2_O, 95%/2.5%/2.5%, rt, 8 h. DIPEA = di*iso*propyl ethylamine, TFA = trifluoroacetic acid.

The same strategy (Scheme 2) was followed for the *iso*DGR cyclic peptidomimetic **10**, using only the (1*R*,2*S*)-*cis*-β-ACPC scaffold and changing the loading sequence of the amino acids on the resin. The cyclic *iso*DGR compound **10** was obtained in an overall 16% yield, with a 99% purity. The overall yields for compounds **8**–**10** were calculated based on the weight and loading of the resin used for the SPPS and the millimoles of the final purified cyclic peptides (i.e., loading of the first amino acid on the resin, all the coupling steps, cleavage, cyclization, final deprotection and purifications). We believe that the cyclization is the crucial step lowering the yields in our case. Conformational preorganization is in general essential for cyclization, and the presence of elements able to impart a turn to the linear peptide chains (especially if located in the central region of the polypeptide), greatly improves the yields [34,35]. In our case this preorganization is missing and the final yields are only satisfying.

### 2.2. Biological Assays

The cyclic peptidomimetics **8**–**10** were analyzed in a first instance for their competitive binding to purified α_v_β_3_ and α_5_β_1_ receptors with respect to the biotinylated vitronectin or fibronectin, respectively. The IC_50_ values are collected in Table 1, where the values of Cilengitide (**1a**), *cyclo*-[RGDfV] (**1b**), *cyclo*-[DKP-RGD] (**4**), and *cyclo*-[DKP-*iso*DGR] (**7**), measured using the same procedure [29,48,49,50], are included as reference compounds for the RGD and *iso*DGR series. The new RGD compounds *cyclo*-[-Arg-Gly-Asp-(1*S*,2*R*)-β-ACPC-Val-] (**8**) and *cyclo*-[-Arg-Gly-Asp-(1*R,*2*S*)-β-ACPC-Val-] (**9**) were found to be nanomolar α_v_β_3_ integrin ligands, with a binding IC_50_ value one order of magnitude higher than **4**. On the other hand, the IC_50_ values measured for α_5_β_1_ were higher than those for α_v_β_3,_ displaying a selectivity ratio (IC_50_α_5_β_1_/IC_50_α_v_β_3_) ranging from about 12 (compound **9**) to 73 (compound **8**), and confirming the trend already observed in the case of ligand **4** [49], On the other hand, the new *iso*DGR ligand *cyclo*-[-*iso*Asp-Gly-Arg-(1*R,*2*S*)-β-ACPC-Val-] (**10**) is a micromolar binder for both integrins, much weaker than *cyclo*-[DKP-*iso*DGR] (**7**) in the case of α_v_β_3_.

In vitro cell adhesion assays were also performed using WM115 cells (a human skin melanoma cell line in which adhesion to ECM proteins is mediated predominantly by integrin α_v_β_3_). The inhibition of the adhesion of the cells was evaluated against plate-coated vitronectin and the IC_50_ values are collected in Table 2. The IC_50_ value of *cyclo*-[RGDfV] (**1b**), measured using the same procedure, is also listed for comparison.

The trend observed in the competitive binding test on the isolated integrin (Table 1) was substantially confirmed in the cell adhesion assay with WM115 cells. Also in this case, despite the different stereochemistry of the two contained *cis*-β-ACPC scaffolds, the two cyclic RGD peptidomimetics (**8** and **9**) showed IC_50_ values in the same micromolar range, one order of magnitude higher than the value of the reference compound (Table 2). Similar to the binding data, the *iso*DGR peptidomimetic **10** is the weakest inhibitor of cell adhesion among the tested compounds (very high micromolar IC_50_ values).

### 2.3. NMR Studies

The structure and connectivity of ligands **8**–**10** were unambiguously assigned by means of mono- and two-dimensional ^1^H and ^13^C NMR spectroscopy in H_2_O solution. The preferred conformations of the cyclic peptidomimetics **8**–**10** were then investigated, with the aim of rationalizing the affinity of these compounds for the α_v_β_3_ receptor at a molecular level. The cyclic peptides are characterized by a high density of carbonyl groups and amide protons close together. In this situation, the formation of hydrogen bonds is favored as well as the presence of β-turn type folds. H-bonds were detected by variable temperature-NMR (VT-NMR) experiments observing the change of NH chemical shift with temperature, while NH_i_-NH_i+1_ NOE contacts, which are indicative of β-turn motifs, were identified by NOESY experiments.

The chemical shifts and temperature coefficients (Δδ/ΔT) of the amide protons relative to compounds **8**–**10** are reported in Table 3. The chemical shift of amide protons not involved in H-bonds generally shows significant temperature dependence (e.g., temperature coefficients in the range −7/−9.5 ppb K^−1^), whereas temperature coefficients between −2 and −5 ppb K^−1^ are indicative of H-bond formation.

The relevant long range NOE contacts, classified as strong or medium on the basis of a qualitative assessment of their intensities, are shown in Table 4. Since these small cyclic molecules can fluctuate between different conformations, NMR data were used to evaluate the most representative 3D structures.

#### 2.3.1. Conformational Analysis of Compound **8**

**Figure 3:**
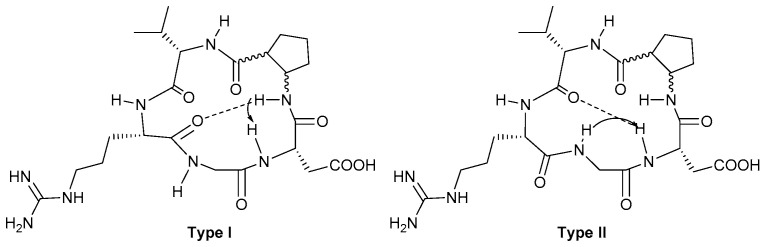
Preferred intramolecular H-bond/β-turn patterns proposed for peptidomimetics **8** and **9** based on spectroscopic data. The arrows indicate significant NOE contacts. Type I H-bonding pattern, Gly-Asp β-turn motif. Type II H-bonding pattern, Arg-Gly β-turn motif (the pattern definition is consistent with that previously reported for other cyclic RGD pentapeptide mimics) [28].

The NMR data of compound **8** suggest that the NH-Asp amide proton (Δδ/ΔT −2 ppb K^−1^, chemical shift 7.73 ppm) is involved in an intramolecular hydrogen bond. In addition, also the Δδ/ΔT of NH-ACPC amide proton (−4 ppb K^−1^) suggests that it is experiencing an intramolecular hydrogen bonding. The other Δδ/ΔT values are in the −6/−9.5 ppb K^−1^ range which is typical of solvent exposed amide protons. In the NOESY spectrum (mixing time 700 ms) two cross peaks involving NH-Asp can be detected: a strong NOE contact between NH-Asp and NH-ACPC, and a medium intensity NOE with NH-Gly. These long range NOEs are exclusive and provide evidence of two preferred conformations characterized by β-turn motifs. The NH-Asp—NH-ACPC NOE is indicative of a β-turn at Gly-Asp (Type I conformation in Figure 3) which is possibly stabilized by a hydrogen bond between NH-ACPC and C=O-Arg. The medium intensity NH-Asp—NH-Gly contact suggests a β-turn at Arg-Gly (Type II conformation in Figure 3) which might be stabilized by a hydrogen bond between NH-Asp and C=O-Val.

#### 2.3.2. Conformational Analysis of Compound **9**

The NMR data of compound **9** point out a large conformational equilibrium, possibly also due to a pseudo-chair ring inversion of the β-ACPC scaffold. A cross-peak of strong intensity between NH-Asp and NH-ACPC is observed in the NOESY spectrum, suggesting that the Type I conformation (Figure 3) might contribute also to the conformational equilibrium of the pseudopeptide **9** (in agreement with the Δδ/ΔT value of NH-ACPC of −5 ppb K^−1^) [51].

#### 2.3.3. Conformational Analysis of Compound **10**

**Figure 4:**
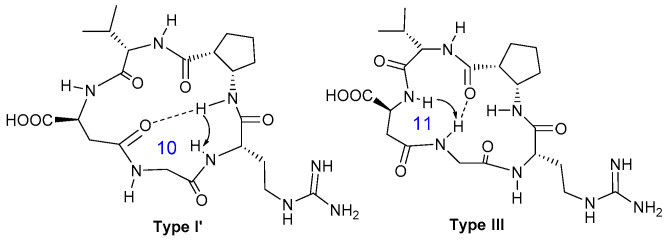
Preferred intramolecular H-bond/β-turn patterns proposed for the *iso*DGR cyclopeptide **10** spectroscopic data. The arrows indicate significant NOE contacts. Type I’ H-bonding pattern, Gly-Arg β-turn motif. Type III H-bonding pattern, Val-*iso*Asp pseudo β-turn motif.

The chemical shift and the Δδ/ΔT values of the amide protons of the isoDGR derivative **10** (Table 3) indicate that NH-Gly and NH-ACPC are involved in hydrogen bonds. Their values suggest the presence of an equilibrium between conformations in which these protons either form intramolecular hydrogen bonds or they are exposed to the solvent, forming hydrogen bonds with it. The other amide protons experience only H-bonds with the solvent. Two mutually exclusive long range NOE contacts are indicative of two different conformations, hereafter referred to as Type I’ and Type III conformations (Figure 4). The strong cross-peak between NH-Arg and NH-ACPC is consistent with a β-turn motif at Gly-Arg, which might be stabilized by a hydrogen bond between NH-ACPC and C=O-*iso*Asp (Figure 4). This Type I’ H-bond/β-turn pattern suggested for the *iso*DGR cyclopeptide **10** is very similar to the Type I pattern defined for the RGD derivatives **8** and **9** (Figure 3). The medium dipolar interaction between NH-Gly and NH-*iso*Asp detected in the NOESY spectrum of compound **10** suggests the presence of a pseudo β-turn at Val-*iso*Asp, which could be stabilized by a H-bond between NH-Gly and C=O-ACPC through the formation of a 11-membered ring (Type III, Figure 4).

### 2.4. Computational Studies

The conformation and the interaction with the α_v_β_3_ integrin of the cyclic peptidomimetics **8**–**10** were investigated by means of computational studies to generate models fitting spectroscopic and biological data.

Monte Carlo/Energy Minimization (MC/EM) conformational searches [52] of simplified cyclic pentapeptide analogs (containing β-ACPC and methyl groups in place of Val, Arg and Asp/*iso*Asp side chains), followed by molecular dynamics (MD) simulations of the RGD or *iso*DGR peptidomimetic ligands, were run in water, as implicitly represented by the generalized Born/surface area (GB/SA) solvation model [53]. The compounds displayed high flexibility, by adopting different backbone geometries characterized by specific H-bond and β-turn patterns. Notably, the macrocycle conformations found for the new ligands are very similar to the geometries previously detected for other cyclic RGD pentapeptide mimics [28]. The analysis of the dihedral angle between the amino and the carboxylic group in the β-ACPC scaffold revealed absolute values of about 60°, with a preference for negative or positive values that depend on the stereochemistry of the *cis*-β-ACPC unit (i.e., preferred negative values for compound **8**, positive values for compound **9**). The inversion of the pseudo-chair ring conformation of the β-ACPC scaffold was indeed observed in the calculated structures of each compound, even if rarely and independently from the macrocycle conformation.

Among the conformers within 3 kcal/mol of the global minimum calculated for the (β-ACPC-Ala-Ala-Gly-Ala) analog of **8**, cyclopeptide geometries were identified that nicely fit the Type I and II patterns suggested on the basis of the NMR data (Figure 3). Three-dimensional structures satisfying the characteristic NOE contact and H-bond of each pattern were selected from the MD trajectory of ligand **8** and then employed as starting geometries for docking studies in the α_V_β_3_ integrin active site (see the Experimental section for computational details) [54]. The best pose of each pattern was re-docked to generate optimized poses that were compared to the crystal structure of the cyclic pentapeptide Cilengitide in complex with the extracellular segment of integrin α_v_β_3_ (PDB code 1L5G).

**Figure 5:**
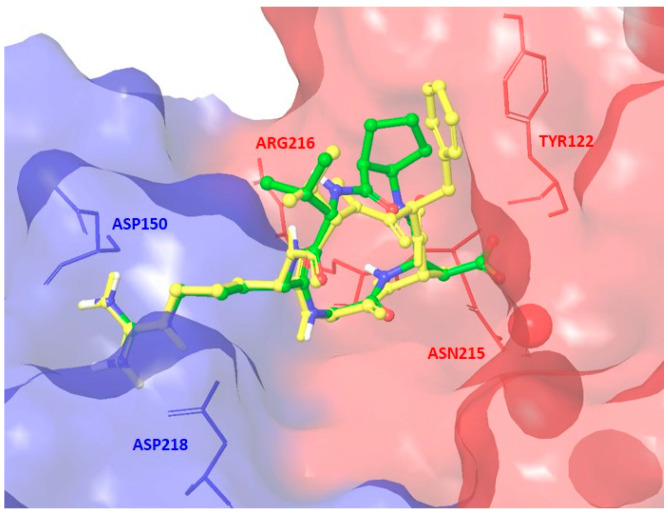
Docking best pose of compound **8** (Type I, green C atoms, Gscore = −9.26 kcal/mol) in the crystal structure of the extracellular domain of α_v_β_3_ integrin (α unit blue, β unit red) overlaid on the bound conformation of Cilengitide (yellow). Only selected integrin residues involved in interactions with the ligand are shown. The metal ion at MIDAS is shown as a red CPK sphere (space-filling model by Corey, Pauling, Koltun). Nonpolar hydrogens are hidden for clarity.

Docking runs starting from geometries of **8** adopting the Type I pattern produced top-ranked poses conserving most of the key interactions observed in the X-ray complex. In the best pose shown in Figure 5, the positively charged Arg guanidinium group of ligand **8** interacts with the negatively charged side chains of Asp218 and Asp150 in the α unit, one carboxylate oxygen of the ligand Asp side chain is coordinated to the metal cation in the metal-ion-dependent adhesion site (MIDAS) of the β unit, and the second carboxylate oxygen forms hydrogen bonds with the backbone amides of Asn215 and Tyr122 in the β unit. However, several other docking poses, including those obtained from Type II starting geometries (see the Supporting Information for the docking best pose of Type II conformation), lose some H-bond interactions or poorly reproduce the crystallographic binding mode mainly interacting with the β-subunit.

A possible explanation of this behavior may be found in the lack of the aromatic moiety and of the corresponding stabilizing interaction with the side chain of Tyr122 in the β subunit, as well as in the less extended arrangement of the RGD sequence in Type II conformations that is known to prevent the optimal binding with the integrin pocket. Indeed, in agreement with previous studies on different cyclic RGD pentapeptide mimics, Type I conformations are characterized by extended arrangements of the recognition motif (showing Cβ(Arg)–Cβ(Asp) distance values of about 9 Å), while the β-turn motif at Arg-Gly of the Type II pattern induces a reduced extension of the RGD sequence (showing Cβ(Arg)–Cβ(Asp) distance values of about 8 Å) [28].

In summary, although peptidomimetic **8** can adopt extended conformations of the recognition motif that should be able to properly fit into the receptor active site and establish the key polar interactions, the absence of an aromatic group and the conformational flexibility of the cyclopeptide induce a notable mobility of the ligand in the integrin binding pocket. This fact is possibly responsible for the non-optimal interaction pattern and of the IC_50_ value in competitive binding assay to α_V_β_3_ receptor about 10 times higher than that of other cyclic RGD ligands (e.g., compound **4** in Table 1).

Similarly, different cyclopeptide geometries can be identified for compound **9** by means of MC/EM conformational searches and MD simulations, including geometries that nicely fit the Type I pattern suggested on the basis of the NMR data (Figure 3). Docking calculations (see the Experimental section for computational details and the Supporting Information for the docking best pose) showed that the conformations adopting this pattern produced top-ranked poses conserving most of the key interactions observed in the X-ray complex. However, the non-extended arrangement adopted by the RGD sequence in some other geometries prevents an optimal interaction with the integrin pocket. Similar considerations to those made for the compound **8** might explain the binding affinity of ligand **9**: the lack of an aromatic moiety and the conformational flexibility of the cyclopeptide make the interaction and the geometrical preorganization less favorable than in other cyclic peptidomimetic RGD ligands.

Finally, docking studies of the *iso*DGR peptidomimetic **10** revealed that both Type I’ and Type III geometries are not able to fit unhindered the α_V_β_3_ binding site, producing non-optimal binding modes (as shown in the Supplementary Materials).

## 3. Material and Methods

### 3.1. General Procedures

The synthesis of (1*S*,2*R*)-*cis*-2-amino-1-cyclopentanecarboxylic acid and (1*R*,2*S*)-*cis*-2-amino-1-cyclopentanecarboxylic acid were performed following published procedures [55,56]. The synthetic procedures for the preparation of compounds **8**–**10** and their characterization are reported in the Supporting Information, along with the ^1^H NMR and ^13^C NMR spectra, HPLC traces.

### 3.2. Competitive Binding Assays to the Purified αvβ3 and α5β1 Receptors

The inhibition assays of biotinylated vitronectin and fibronectin binding to the αvβ3 and α5β1 receptors, for compounds **8**–**10** were carried out as previously reported [29,48,49,50]. IC_50_ values were calculated as the concentration of compound required for 50% inhibition of biotinylated vitronectin or fibronectin binding. Screening assays were performed by incubating the immobilized integrin αvβ3 or α5β1 with increasing concentrations (10^−12^–10^−5^ M/10^−11^–10^−4^ M) of the RGD and isoDGR ligands in the presence of the corresponding biotinylated ECM protein (1 μg mL^−1^), and measuring bound protein in the presence of the competitive ligand. Each data point is the result of the average of triplicate wells and was analyzed by nonlinear regression analysis with the GraphPad Prism software. Each experiment was repeated in duplicate. IC_50_ values are the arithmetic mean ± the standard deviation (SD) of these duplicate determinations.

### 3.3. Cell Adhesion Assays

The cell adhesion studies on WM115 cell line with the compounds **8**–**10** are described in the Supporting Information.

### 3.4. NMR Studies

NMR experiments were performed on a Bruker Avance spectrometer (Bruker, Málaga, Spain) operating at 500 MHz at 298 K. The concentration of each compounds was 5 mM and they are analyzed dissolved in H_2_O-D_2_O 9:1 in a 5 mm NMR tube.

^1^H and ^13^C resonance assignment for all the ligands was performed on the base of 1D ^1^H, 2D COSY, TOCSY and ^1^H, ^13^C-HSQC experiments. For conformational analysis, NOESY experiments were acquired varying the mixing time from 400 to 700 ms. Water suppression was achieved by excitation sculpting sequence from standard Bruker library.

For the variable temperature analysis (VT-NMR), monodimensional ^1^H spectra were acquired from 298 K to 333 K.

### 3.5. Computational Studies

All calculations were performed using the Schrödinger suite of programs through the Maestro graphical interface [57]. Conformational preferences of compounds were investigated by molecular mechanics calculations using the MacroModel v11.1 implementation of the Amber all-atom force field (denoted AMBER *) and the implicit water GB/SA solvation model [53,58]. Monte Carlo/energy minimization (MC/EM) conformational search of the cyclopeptide analog containing methyl groups instead of the Val, Arg and Asp/*iso*Asp side chains was performed as the first step to generate starting cyclopeptide conformations for MD simulations that are not biased by electrostatic interactions. For the search, 1000 starting structures for each variable torsion angle were generated and minimized until the gradient was < 0.05 kJ Å^−1^ mol^−1^ using the truncated Newton-Raphson method implemented in MacroModel [59]. Duplicate conformations and those with energy > 5 kcal mol^−1^ above the global minimum were discarded. Free MD simulations of the RGD cyclic peptides (Asp and Arg side chains were considered ionized) were then performed at 300 K using MacroModel v11.1, the force field and the solvent defined above (1 fs integration step, 20 ns simulation time for each run, 5000 structures saved for the analysis), starting from the cyclopeptide backbone geometries located by the previous MC/EM step. Each significant long range interaction between amide protons described by a NOE was employed as a filter (distance between protons involved in the NOE contact < 3.5 Å) to select the conformations fitting a specific experimental contact. Representative 3D structures filtered out from MD trajectories and reproducing the H-bond patterns pointed out by NMR data, were employed as starting geometries for docking studies.

The crystal structure of the extracellular domain of the integrin α_v_β_3_ in complex with the cyclic pentapeptide Cilengitide (PDB code 1L5G) was used for docking studies [60]. Flexible-ligand docking calculations were performed using Glide version 7.0 in the Standard Precision (SP) mode [61]. The settings of the protein preparation, grid generation and docking step were defined as previously reported [27,28,29]. The Glide program was initially tested for its ability to reproduce the X-ray binding geometry of Cilengitide. The program was successful in reproducing the experimentally found binding mode of this compound, as it corresponds to the best-scored pose (the superimposition of the docked ligand to the crystal structure is shown in the Supporting Information, docked vs. X-ray peptide heavy atoms RMSD = 0.3287 Å).

## 4. Conclusions

The synthesis, conformational analysis, and some biological investigation of two RGD, **8**–**9** (Figure 2), and one *iso*DGR, **10** (Figure 2) cyclic peptidomimetics, containing two *cis*-2-amino-1-cyclopentanecarboxylic (either (1*S*,2*R*)-*cis*-β-ACPC or (1*R*,2*S*)-*cis*-β-ACPC) scaffolds were described. The synthesis of the linear precursors was performed on the solid phase and, after cleavage from the resin and cyclization, the integrin ligands were obtained in satisfying yields and excellent purity. The RGD compounds **8** and **9** are good α_v_β_3_ integrin ligands, confirming this behavior both in an isolated receptor competitive binding assay (IC_50_ values 44 and 39 nM, respectively, one order of magnitude higher than the reference compound *cyclo*-[RGDfV]), and in the cell adhesion assay performed on the α_V_β_3_ positive human skin melanoma cell line WM115 (IC_50_ values 75 and 124 μM, respectively), again one order of magnitude higher than the reference compound *cyclo*-[RGDfV]). The ligands were also tested for competitive binding to purified α_5_β_1_ giving IC_50_ values above 450 nM, thus confirming the trend, albeit with a selectivity ratio (IC_50_α_5_β_1_/IC_50_α_v_β_3_) ranging from about 12 (compound **9**) to 73 (compound **8**). On the contrary, the *iso*DGR ligand **10** is a micromolar for both α_v_β_3_ and α_5_β_1_ binder (5362 and 2331 nM respectively) in the competitive binding assay and an IC_50_ value could not be measured for the cell adhesion assay. The behavior of these ligands could be explained by the conformational NMR and computational studies of the ligands and the docking simulations performed in the α_V_β_3_ integrin active site. The RGD ligands display intramolecular hydrogen bonds imposing well defined conformations and extended presentation of the RGD recognition motif that should be able to properly fit into the receptor active site and establish the key polar interactions. However, the non-extended arrangement adopted by the RGD sequence in other geometries as well as the absence of an aromatic moiety and of the corresponding stabilizing interaction with the side chain of Tyr122 in the β subunit, prevent an optimal interaction with the integrin pocket and reduce the activity of these ligands as integrin binders. On the other hand, the geometries adopted by the *iso*DGR ligand **10** are not able to fit the α_v_β_3_ binding site, producing non-optimal binding modes.

Further studies aimed at improving the activity and selectivity of these ligands (based on a rational design and plan to modification of the structure of the peptidomimetic scaffold) are currently in progress in our laboratories.

## Figures and Tables

**Table 1:** IC_50_ values measured from competitive binding assays to α_v_β_3_ and α_5_β_1_ integrins.

Compound	α_v_β_3_ IC_50_ [nM] ^[a]^	α_5_β_1_ IC_50_ [nM] ^[a]^
Cilengitide (**1a**)	0.71 ± 0.06	14.4 ± 3.1
*cyclo*-[-Arg-Gly-Asp-d-Phe-Val-] (**1b**)	3.2 ± 1.3	166 ± 28
*cyclo*-[-DKP-Arg-Gly-Asp-] (**4**)	4.5 ± 1.1	532 ± 35
*cyclo*-[-Arg-Gly-Asp-(1*S*,2*R*)-β-ACPC-Val-] (**8**)	44.3 ± 4.0	3227 ± 1468
*cyclo*-[-Arg-Gly-Asp-(1*R,*2*S*)-β-ACPC-Val-] (**9**)	39.0 ± 1.1	468 ± 114
*cyclo*-(-DKP-*iso*Asp-Gly-Arg-) (**7**)	9.2 ± 1.1	1066 ± 228
*cyclo*-[-*iso*Asp-Gly-Arg-(1*R,*2*S*)-β-ACPC-Val-] (**10**)	5362 ± 281	2331 ± 134

^[a]^ IC_50_ values were calculated as the concentration of compound required for 50% inhibition of biotinylated vitronectin or fibronectin binding. Screening assays were performed by incubating the immobilized integrin α_v_β_3_ or α_5_β_1_ with increasing concentrations (10^−12^–10^−5^ M/10^−11^–10^−4^ M) of the RGD and *iso*DGR ligands in the presence of the corresponding biotinylated ECM (extracellular matrix) protein (1 μg mL^−1^), and measuring bound protein in the presence of the competitive ligand. Each data point is the result of the average of triplicate wells and was analyzed by nonlinear regression analysis with the GraphPad Prism software. Each experiment was repeated in duplicate. All values shown are the arithmetic mean ± the standard deviation (SD) of these duplicate determinations.

**Table 2:** IC_50_ values of the cyclic peptides as determined by cell adhesion assays with WM115 cells.

Compound	IC_50_ [μM] ^[a]^
*cyclo*-(-Arg-Gly-Asp-d-Phe-Val-) (**1b**)	5.1 ± 1.8
*cyclo*-[-Arg-Gly-Asp-(1*S*,2*R*)-β-ACPC-Val-] (**8**)	75 ± 32.5
*cyclo*-[-Arg-Gly-Asp-(1*R*,2*S*)-β-ACPC-Val-] (**9**)	124.5 ± 10.6
*cyclo*-[-*iso*Asp-Gly-Arg-(1*R*,2*S*)-β-ACPC-Val-] (**10**)	>300

^[a]^ IC_50_ values were calculated as the concentration of compound required for 50% inhibition of cell adhesion to vitronectin. Cell adhesion assays were performed by incubating the immobilized vitronectin with fluorescein labeled cells and increasing concentrations (10^−7^–10^−4^ M) of the RGD and *iso*DGR ligands, and measuring the fluorescence of bound cells. All values are the arithmetic mean ± the standard deviation (SD) of two independent assays each with four replicate determinations.

**Table 3:** Chemical shifts (δ, ppm) and temperature coefficients (Δδ/ΔT, ppb K^−1^) of amide protons of peptidomimetics **8**–**10** in H_2_O solution.

Compound	NH-Gly δ (Δδ/ΔT) ^[a]^	NH-Arg δ (Δδ/ΔT) ^[a]^	NH-Asp δ (Δδ/ΔT) ^[a]^	NH-ACPC δ (Δδ/ΔT) ^[a]^	NH-Val δ (Δδ/ΔT) ^[a]^
**8**	8.37 (−7)	9.10 (−9.5)	7.73 (−2)	7.33 (−4)	7.92 (−6)
**9**	8.62 (−7)	8.30 (−5)	8.38 (−8)	7.59 (−5)	7.52 (−6)
**10**	8.04 (−3)	8.44 (−7)	8.42 (−7)	7.91 (−4)	7.74 (−9)

^[a]^ The temperature variable experiments were performed in the range of 298–328 K.

**Table 4:** Relevant long range NOE contacts for compounds **8–10**.

Compound	NOE Contact ^[a]^	NOE Contact ^[a]^
**8**	NH-Asp—NH-ACPC (s)	NH-Gly—NH-Asp (m)
**9**	NH-Asp—NH-ACPC (s)	
**10**	NH-Arg—NH-ACPC (s)	NH-Gly—NH-*iso*Asp (m)

^[a]^ s = strong, m = medium.

## Supplementary Materials

Detailed synthetic procedures and characterization for compounds 8–10. Figures S1–S3: HPLC traces of compounds 8–10. Biological Tests (cell culture, determination of IC50 values, cell adhesion experiments). Figures S4 and S5: Cell adhesion curves for compounds 8–10. Computational studies, docking calculations. Figures S6–S9: Docking best poses for Cilengitide and compounds 8–10.
