# Supplementary material for: Cyclic RGD and isoDGR Integrin Ligands Containing cis-2-amino-1-cyclopentanecarboxylic (cis-β-ACPC) Scaffolds
Source: Molecules. 2020 Dec 16;25(24):5966. doi: 10.3390/molecules25245966 (PMC7766232; doi:10.3390/molecules25245966)
Supplement: Supplementary file 1 [file molecules-25-05966-s001.pdf]

Supporting Information

Cyclic RGD and *iso*DGR Integrin Ligands Containing *cis*-2-amino-1-cyclopentanecarboxylic (*cis*-β-ACPC) Scaffolds

Silvia Panzeri [1,5], Daniela Arosio [2], Silvia Gazzola [1], Laura Belvisi [3], Monica Civera [3], Donatella Potenza [3], Francesca Vasile [3], Isabell Kemker [4], Thomas Ertl [5], Norbert Sewald [4], Oliver Reiser [5] and Umberto Piarulli [1,*]

[1] Dipartimento di Scienza e Alta Tecnologia, Università degli Studi dell'Insubria, Via Valleggio 11, 22100 Como, Italy; silvia.panzeri.87@gmail.com (S.P.); s.gazzola@uninsubria.it (S.G.)

[2] Consiglio Nazionale delle Ricerche (CNR), Istituto di Scienze e Tecnologie Chimiche (SCITEC), Giulio Natta, Via C. Golgi 19, 20133 Milan, Italy; daniela.arosio@scitec.cnr.it

[3] Dipartimento di Chimica, Università degli Studi di Milano, Via C. Golgi 19, 20133 Milan, Italy; laura.belvisi@unimi.it (L.B.); monica.civera@unimi.it (M.C.); donatella.potenza@unimi.it (D.P.); francesca.vasile@unimi.it (F.V.)

[4] Department of Chemistry, Organic and Bioorganic Chemistry, University of Bielefeld, Universitätsstraße 25, DE-33615 Bielefeld, Germany; isabell.kemker@uni-bielefeld.de (I.K.); norbert.sewald@uni-bielefeld.de (N.S.)

[5] Institute of Organic Chemistry, University of Regensburg, Universitätsstraße 31, 93053 Regensburg, Germany; thomas.ertl@chemie.uni-regensburg.de (T.E.); Oliver.Reiser@chemie.uni-regensburg.de (O.R.)

* Correspondence: Umberto.Piarulli@uninsubria.it

Academic Editor: Pierangela Ciuffreda
Received: 24 October 2020; Accepted: 14 December 2020; Published: 16 December 2020

Materials and methods

All commercially available reagents were used as received; DMF was anhydrified according to **GP0**. Non solid-phase reactions were monitored by analytical thin layer chromatography (TLC) using silica gel 60 F_{254} pre-coated glass plates (0.20 mm thickness). Visualization was accomplished by irradiation with a UV lamp and/or staining with a potassium permanganate alkaline solution or with a ninhydrin solution. Solid phase steps was followed by LR-Mass. Flash column chromatography was performed according to the method of Still and co-workers[1] using Chromagel 60 ACC (40–63 m) silica gel. Proton NMR spectra were recorded on a spectrometer operating at 500 MHz. Carbon NMR spectra were recorded on a spectrometer operating at 125 MHz, with complete proton decoupling. The following abbreviations are used to describe spin multiplicity s = singlet, d = doublet, t = triplet, q = quartet, m = multiplet, bs = broad signal, dd = doublet of doublet, ddd = doublet of doublet of doublet, ddt = doublet of doublet of triplet. Carbon chemical shifts are reported in ppm relative to TMS with the respective solvent resonance as the internal standard. ESI-MS spectra were recorded on the ion trap mass spectrometer Finnigan LCQ Advantage.

General procedures for SPPS

DMF anhydrification

GP0: A flask was charged with CaH_2 (around 5 g/L) and DMF. The mixture was stirred at room temperature overnight. DMF was subsequently transferred into a flask under N_2 and distilled under reduced pressure (around 20 mBar). DMF was stored under N_2 in a dark brown flask over molecular sieves (3Å).

[1] Still, W. C.; Kahn, M.; Mitra, A., *J. Org. Chem.* **1978**, *43*, 2923-2925.

SPPS was manually accomplished using a shaker. Fmoc strategy and 2-chlorotritylchloride resin (100-200 mesh, 1% DVB; loading: 1.51 mmol/g resin) were used. Each coupling step consisted in:

i) Activation of the Fmoc-protected amino acid,

ii) Addition of the activated amino acid to the resin at the shaker to effect the coupling reaction;

iii) Following steps of capping, deprotection and washing.

Resin preparation and storage

The resin was weighted in a 10 mL vial and swelled (**GP1**); at the end of this operation, the resin was ready for the SPPS. When necessary, beads were stored at 4 °C under nitrogen after being washed several times with DCM and dried at the high vacuum pump.

Swelling of the resin

GP1: The resin was weighted in a vial, suspended in DCM (approximately 10 mL per gram of resin) and shaken for 30 min. The solvent was dried from the resin and, at the end of this operation, the resin was ready for the SPPS. This step was repeated every time before starting all the procedures on the resin (if the resin was previously dried under vacuum and stored at 4°C).

Loading of the first amino acid and capping of the resin

GP2: The resin was suspended in DCM (approximately 14 mL per gram of resin); Fmoc-AA-OH (2.0 equiv.) and DIPEA (0.3 equiv.) were sequentially added. The resin was shaken for 10 min then DIPEA (0.5 equiv.) was added and the resin was shaken for 1h 30min; after this time the solvent was removed. The resin was washed with DCM (3 × 2 mL), then methanol was added (approximately 1.5 mL per gram of resin) and the resin was shaken for 15 min. The solvent was removed and the resin was washed with DCM (3 × 2 mL), DMF (3 × 2 mL), DCM again (3 × 2 mL), and methanol (1 × 2 mL).

Amino acid activation and coupling

GP3: To a 0.14 M solution of the amino acid (1.5 equiv.) in dry DMF under N_2 at 0 °C, TBTU (1.5 equiv. in DMF) and DIPEA (3.0 equiv. in DMF) were added. The mixture was stirred under these conditions for 30-40 min and then the solution was added to the resin. The resin was shaken at r.t. for 3 h then the solvent was removed. Each coupling step was performed twice times. After the second coupling, the resin was washed with DCM (3 × 2 mL), DMF (3 × 2 mL), and DCM again (3 × 2 mL).

Scaffold activation and coupling

GP4: To a solution of the scaffold (1.5 equiv.) in dry DMF (solution 0.14 M) under N_2 at 0 °C, HATU (1.5 equiv. in DMF) and DIPEA (3.0 equiv. in DMF) were added. The mixture was stirred under these conditions for 45 min and then the solution was added to the resin. The resin was shaken at r.t. for 7 h then the solvent was removed. After the coupling, the resin was washed with DCM (3 × 2 mL), DMF (3 × 2 mL), and DCM again (3 × 2 mL).

Capping procedure

GP5: A 1M solution of Ac_2O (50 equiv.) and pyridine (50 equiv.) in DMF (approximately 30 mL per gram of unsubstituted resin) was added to the resin. The mixture was shaken at r.t. for 40 min then solution was removed and beads were washed with DMF (3 × 2 mL).

Fmoc removal procedure

GP6: Deprotection of the *N*-terminal Fmoc group was achieved using a solution of 2% DBU and 2% piperidine (*v*/*v*) in DMF (approximately 30 mL per gram of the unsusbstituted resin). The solution was added to the resin that was shaken for 15 min then the solution was removed. Two deprotection steps were carried out. At the end of each step, the solvent was removed. At the end of the second step, beads were sequentially washed with DMF (6 × 2 mL) and DCM (3 × 2 mL).

Peptide cleavage:

GP7: The resin was washed with DCM (3 × 2 mL) and then treated with 1% trifluoroacetic acid in DCM (15.0 mL per gram of the not substituted resin) for 5 min each time. Treatment was repeated as long as the peptide was cleaved from the resin (approximately 10 times), checking by TLC (staining with a ninhydrin solution). Combined solutions were evaporated under reduced pressure and then in vacuum. The crude obtained was used without purification in the macrolactamization step.

General procedures for solution-phase synthesis

Macrolactamization

GP8: The linear precursor (1 equiv.) was dissolved in dry DMF (5 mM solution) and transferred into a syringe. HATU (3.0 equiv.) was dissolved in the same volume of dry DMF and transferred into a second syringe. These two solutions were added slowly using two syringe pumps to a stirred solution of DIPEA (6.0 equiv.) and HATU (0.1 equiv.) in half of the previous volume of dry DMF at a rate of 0.7mL/h. Once the addition was complete, the mixture was stirred for 30 min. The solvent was evaporated under reduced pressure at a temperature lower than 35°C. Crude was taken up with ethyl acetate (25 mL), sequentially washed with a solution 1M of $KHSO_4$, a saturated solution of $NaHCO_3$ and brine. The organic layer was dried over Na_2SO_4 anhydrous and volatiles were removed under reduced pressure. The crude dissolved in DCM:MeOH (9:1) and filtered over silica gel.

Side chain deprotection

GP9: The crude fully protected macrolactam was treated for 8 h with a solution (around 21 mL/mg of protected peptide) of trifluoroacetic acid (95%) at room temperature in the presence of water (2.5%) and triisopropylsilane (2.5%). After volatiles removal under reduced pressure, the residue was taken up with a 1:1 mixture of diethyl ether/water. Phases were separated and the aqueous layer was washed several times with diethyl ether. The aqueous phase was concentrated under reduced pressure and then the solution was freeze-dried. The crude was purified by preparative HPLC.

RP-HPLC purification methods

For RGD compounds (**8** and **9**), HPLC purifications were performed on an Agilent1100 Series (column: Phenomenex Luna 10 um C18(2) 100A 250 × 21.2 mm). The crude reaction mixture was dissolved in water and the solution was filtered (polypropylene, 0.45 m, 13 mm ø, PK/100) and injected in the HPLC, affording purified products.

For the *iso*DGR compound (**10**), HPLC purifications were performed on a Dionex Ultimate 3000 instrument equipped with a Dionex RS Variable Wavelength Detector (column: Atlantis Prep T3 OBDTM 5 TM 19 x 100 mm). The crude reaction mixture was dissolved in water/Acetonitrile (7:3) and the solution was filtered (polypropylene, 0.45 m, 13 mm ø, PK/100) and injected in the HPLC, affording the purified product.

RP-HPLC analysis

Purity analysis for each of the compounds was carried out on a Dionex Ultimate 3000 instrument equipped with a Dionex RS Variable Wavelength Detector (column: Atlantis Prep T3

OBDTM 5 TM 19 x 100 mm). 0.8 mg of purified product was dissolved in 0.5 mL of H_2O and 0.2 mL of acetonitrile and was injected using gradient: 100% H_2O + 0.1% CF_3COOH/0% CH_3CN + 0.1% CF_3COOH to 50% H_2O + 0.1% CF_3COOH/50% CH_3CN + 0.1% CF_3COOH in 11 min. The analysis of the integrals and the relative percentage of purity was performed with the software Cromeleon 6.80 SR11 Build 3161.

Freeze-drying

Each product was dissolved in water and frozen with dry ice: the freeze-drying was carried out at least for 48 h at −50 °C using the instrument 5Pascal Lio5P DGT.

Preparation of compound 8, 9 and 10

Synthesis of 8 and 9

(1*S*,2*R*)- -ACPC and (1*R*,2*S*)- -ACPC were synthesized according to literature procedures ((a) Bunnage, M. E.; Davies, S. G.; Roberts, P. M.; Smith, A. D.; Withey, J. M. *Org. Biomol. Chem.* **2004**, *2*, 2763; (b) Davies, S. G.; Ichihara, O.; Lenoir, I.; Walters, I. A. S. *J. Chem. Soc., Perkin Trans. 1* **1994**, *11*, 1411).

Asp(O*t*Bu)-(1*S*,2*R*)- -ACPC-Val-Arg(Pbf)-Gly-OH (13) and Asp(O*t*Bu)-(1*R*,2*S*)- -ACPC-Val-Arg(Pbf)-Gly-OH (14).

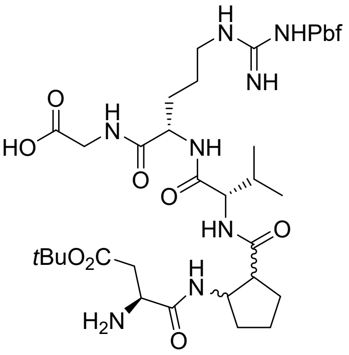

13, 14

The resin was weighed in a solid phase syringe and swelled (**GP1**). Fmoc-Gly-OH was activated and coupled to the resin (**GP2**). After a capping step (**GP5**) and a Fmoc deprotection step (**GP6**), Fmoc-Arg(Pbf)-OH was activated and coupled to the Gly-resin (**GP3**). Then a capping step (**GP5**) and a Fmoc deprotection step (**GP6**) were performed. Fmoc-Val-OH was activated and coupled to the Arg(Pbf)-Gly-resin (**GP3**); the coupling was followed by a capping step (**GP5**) and a Fmoc deprotection step (**GP6**). (1*R*,2*S*)-β-ACPC and (1*S*,2*R*)-β-ACPC were activated and coupled to the Val-Arg(Pbf)-Gly-resin (**GP4**), then a capping step (**GP5**) and a Fmoc deprotection step (**GP6**) were performed. Fmoc-Asp(O*t*Bu)-OH was activated and coupled to the (1*R*,2*S*)-β-ACPC-Val-Arg(Pbf)-Gly-resin (**GP3**) and to the (1*S*,2*R*)-β-ACPC-Val-Arg(Pbf)-Gly-resin (**GP3**), then Fmoc deprotection step (**GP6**) was performed. Linear precursors **11** and **12** were cleaved from the resin (**GP7**), checked by LR-Mass and used in the following step as crudes. Crudes **13** (185 mg) and **14** (140 mg) were obtained as brown oils.

Exact amounts of the amino acids and coupling reagents used for SPPS are reported in in Table S1 for **13** and in Table S2 for **14**.

Table S1.

Reagents	Equiv. or Concentration	mmol	Amounts
Chlorotritylchloride resin (1.51 mmol/g)	1.0 equiv.	0.190	130 mg

Fmoc-Gly-OH*	2.0 equiv.	0.380	113 mg
DIPEA	0.3 equiv.	0.057	10 µL
DIPEA	0.5 equiv.	0.095	17 µL
DCM	/	/	2.0 mL
Fmoc-Arg(Pbf)-OH*	1.5 equiv.	0.285	185 mg
TBTU	1.5 equiv.	0.285	92 mg
DIPEA	3.0 equiv.	0.570	99 µL
DMF	0.14 M	/	2 mL
Fmoc-Val-OH *	1.5 equiv.	0.285	97 mg
TBTU	1.5 equiv.	0.285	92 mg
DIPEA	3.0 equiv.	0.570	99 µL
DMF	0.14 M	/	2 mL
(1*R*,2*S*)-β-ACPC	1.5 equiv.	0.285	100
HATU	1.5 equiv.	0.285	108
DIPEA	4.0 equiv.	0.760	132 µL
DMF	0.14 M	/	2 mL
Fmoc-Asp(O*t*Bu)-OH*	1.5 equiv.	0.285	117 mg
TBTU	1.5 equiv.	0.285	92 mg
DIPEA	3.0 equiv.	0.570	99 µL
DMF	0.14 M	/	2 mL

* a double coupling step was performed.

Table S2.

Reagents	Equiv. or Concentration	mmol	Amounts
Chlorotritylchloride resin (1.51 mmol/g)	1.0 equiv.	0.190	130 mg
Fmoc-Gly-OH*	2.0 equiv.	0.380	113 mg
DIPEA	0.3 equiv.	0.057	10 μL
DIPEA	0.5 equiv.	0.095	17 μL
DCM	/	/	2.0 mL
Fmoc-Arg(Pbf)-OH*	1.5 equiv.	0.285	185 mg
TBTU	1.5 equiv.	0.285	92 mg
DIPEA	3.0 equiv.	0.570	99 μL
DMF	0.14 M	/	2 mL
Fmoc-Val-OH*	1.5 equiv.	0.285	97 mg
TBTU	1.5 equiv.	0.285	92 mg
DIPEA	3.0 equiv.	0.570	99 μL
DMF	0.14 M	/	2 mL
(1*S*,2*R*)-β-ACPC	1.5 equiv.	0.285	100 mg
HATU	1.5 equiv.	0.285	108 mg
DIPEA	4.0 equiv.	0.760	132 μL
DMF	0.14 M	/	2 mL
Fmoc-Asp(O*t*Bu)-OH *	1.5 equiv.	0.285	117 mg
TBTU	1.5 equiv.	0.285	92 mg
DIPEA	3.0 equiv.	0.570	99 μL
DMF	0.14 M	/	2 mL

* a double coupling step was performed.

***Cyclo*-(-Asp(O*t*Bu)-(1*S*,2*R*)- -ACPC-Val-Arg(Pbf)-Gly-) (15) and *cyclo*-(-Asp(O*t*Bu)-(1*R*,2*S*)- -ACPC-Val-Arg(Pbf)-Gly-) (16).**

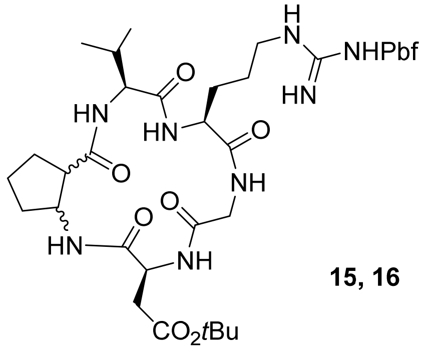

15, 16

Compound **13** and **14** (**13**: 185 mg crude, 0.217 mmol, considered 1.0 equiv. for stoichiometric calculations; **14**: 140 mg crude, 0.163 mmol, considered 1.0 equiv. for stoichiometric calculations) were treated with HATU (**13**: 252 mg, 0.662 mmol, 3.1 equiv.; **14**: 193, 0.489 mmol, 3.1 equiv.) and DIPEA (**13**: 226 µL, 1.299 mmol, 6.0 equiv.; **14**: 170 µL, 0.979 mmol, 6.0 equiv.) under the conditions described in **GP8**. Crude products were obtained as pale yellow solids (**15**: 61 mg, crude; **16**: 94 mg, crude).

***Cyclo*-(-Asp-(1*S*,2*R*)- -ACPC-Val-Arg-Gly-) (8) and *cyclo*-(-Asp-(1*R*,2*S*)- -ACPC-Val-Arg-Gly-) (9).**

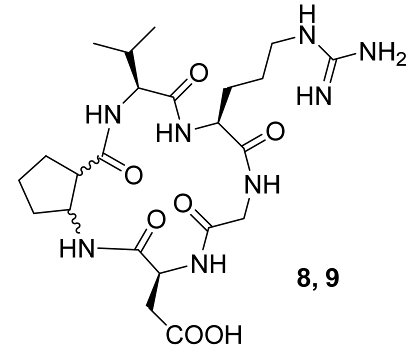

8, 9

Compound **15** (94 mg, crude) and **16** (61 mg, crude) were deprotected under the conditions described in **GP9**. Crude products were purified by HPLC [compound **8** = gradient: 95% H_2O + 0.059% w/w CF_3COOH/5% CH_3CN to 70% H_2O + 0.059% w/w CF_3COOH/30% CH_3CN in 9 min, flow: 21 mL/min; compound **9** = gradient: 95% H_2O + 0.059% w/w CF_3COOH/5% CH_3CN to 70% H_2O + 0.059% w/w CF_3COOH/30% CH_3CN in 9 min, flow: 21 mL/min]. Pure fractions were concentrated and freeze-dried, affording **8** and **9** as white solids (**8**: 13 mg, 0.020 mmol, overall yield 10%; **9**: 11 mg, 0.017 mmol, overall yield 9%).

Compound 8:

^{1}H-NMR (500 MHz, H_2O/D_2O) 9.11 (d, 1H), 8.37 (t, 1H), 7.92 (d, 1H), 7.73 (d, 1H), 7.33 (d, 1H), 7.21 (t, 4H), 4.89 (m, 1H), 4.29 (m, 1H), 4.14-3.68 (m, 2H), 4.12 (m, 1H), 3.86 (m, 1H), 3.23-3.17 (m, 2H), 3.08 (m, 1H), 2.91-2.58 (m, 2H), 2.05 (m, 1H), 1.98-1.81 (m, 2H), 1.91-1.60 (m, 2H), 1.90-1.85 (m, 2H), 1.79-1.58 (m, 2H), 1.78-1.56 (m, 2H), 0.91 (dd, 6H). ^{13}C-NMR (125 MHz, D_2O) 59.2, 54.4, 53.4, 49.6, 46.7, 43.1, 40.6, 36.3, 31.3, 30.4, 27.4, 26.4, 25.9, 22.1, 18.1; MS (ESI) m/z calcd. for $[C_{23}H_{39}N_8O_7]^+$: 539.2936 $[M+H]^+$; found: 539.2935 $[M+H]^+$.

Compound 9:

^{1}H-NMR (500 MHz, H_2O/D_2O) 8.62 (t, 1H), 8.38 (d, 1H), 8.31 (d, 1H), 7.59 (d, 2H), 7.18 (m, 4H), 4.59 (m, 1H), 4.22 (m, 1H), 4.21 (m, 1H), 3.86-3.78 (m, 2H), 3.80 (m, 1H), 3.18 (m, 2H), 2.93-2.86 (m, 2H), 2.92 (m, 1H), 2.09-1.98 (m, 2H), 2.08 (m, 1H), 1.88-1.83 (m, 2H), 1.86-1.80 (m, 2H), 1.80-1.69

(m, 2H), 1.60 (m, 2H), 0.94 (t, 6H). ^{13}C-NMR (125 MHz, D_2O) 61.8, 53.4, 53.4, 50.7, 47.4, 44.1, 40.8, 35.4, 31.1, 28.9, 27.4, 27.1, 25.1, 21.4, 19.0; MS (ESI) m/z calcd. for $[C_{23}H_{39}N_8O_7]^+$: 539.2936 $[M + H]^+$; found: 539.2944 $[M + H]^+$.

Figure S1. HPLC trace of compound 8.

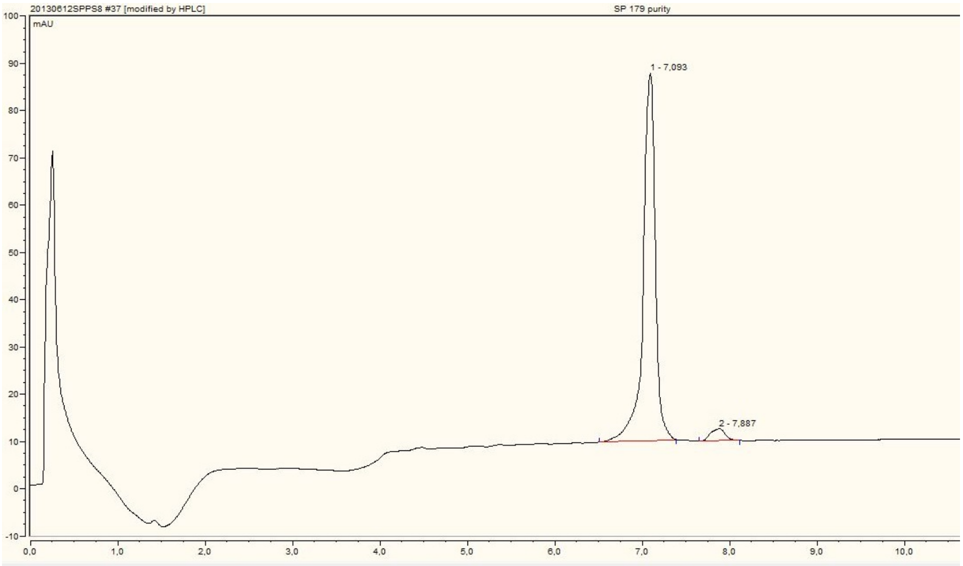

HPLC purity = 96%

Figure S2. HPLC trace of compound 9.

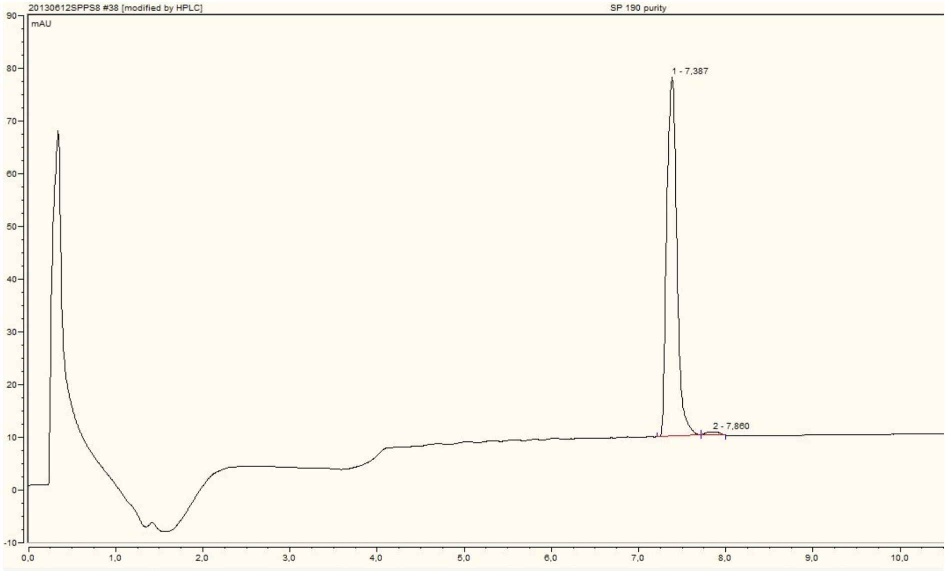

HPLC purity = 99%

Synthesis of 10

Arg(Pbf)-(1*R*,2*S*)- -ACPC-Val-*iso*Asp(O*t*Bu)-Gly-OH (18).

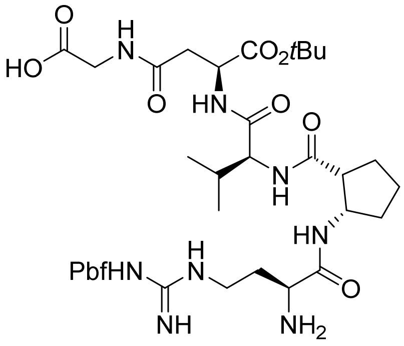

18

The resin was weighed in a solid phase syringe and swelled (**GP1**). Fmoc-Gly-OH was activated and coupled to the resin (**GP2**). After a capping step (**GP5**) and a Fmoc deprotection step (**GP6**), Fmoc-Asp(OH)-O*t*Bu was activated and coupled to the Gly-resin (**GP3**). Then a capping step (**GP5**) and a Fmoc deprotection step (**GP6**) were performed. Fmoc-Val-OH was activated and coupled to the Asp(O*t*Bu)-Gly-resin (**GP3**); the coupling was followed by a capping step (**GP5**) and a Fmoc deprotection step (**GP6**). (1*R*,2*S*)-β-ACPC was activated and coupled to Val-Asp(O*t*Bu)-Gly-resin (**GP4**), then a capping step (**GP5**) and a Fmoc deprotection step (**GP6**) were performed. Fmoc-Arg(Pbf)-OH was activated and coupled to (1*R*,2*S*)-β-ACPC-Val-Asp(O*t*Bu)-Gly-resin (**GP3**), then Fmoc deprotection step (**GP6**) was performed. The linear precursor was cleaved from the resin (**GP7**), checked by LR-Mass and used in the following step as crude. The crude **18** (114 mg) was obtained as a yellow solid.

Exact amounts of the amino acids and coupling reagents used for SPPS are reported in Table S3.

Table S3.

Reagents	Equiv. or Concentration	mmol	Amounts
Chlorotritylchloride resin (1.51 mmol/g)	1.0 equiv.	0.095	65 mg
Fmoc-Gly-OH *	2.0 equiv.	0.190	56 mg
DIPEA	0.3 equiv.	0.029	5 μL
DIPEA	0.5 equiv.	0.048	8 μL
DCM	/	/	1.0 mL
Fmoc-Asp(OH)-O*t*Bu *	1.5 equiv.	0.142	58 mg
TBTU	1.5 equiv.	0.142	46 mg
DIPEA	3.0 equiv.	0.285	50 μL
DMF	0.14 M	/	1.0 mL
Fmoc-Val-OH*	1.5	0.142	48 mg

TBTU	1.5	0.142	46 mg
DIPEA	3.0	0.142	50 μL
DMF	/	/	1.0 mL
(1*R*,2*S*)-β-ACPC	1.5 equiv.	0.142	50 mg
HATU	1.5 equiv.	0.142	54 mg
DIPEA	4.0 equiv.	0.380	66 μL
DMF	0.14 M	/	1.0 mL
Fmoc-Arg(Pbf)-OH *	1.5	0.142	92 mg
TBTU	1.5	0.142	46 mg
DIPEA	3.0	0.285	50 μL
DMF	/	/	1.0 mL

* a double coupling step was performed.

Cyclo-(-Arg(Pbf)-(1R,2S)- -ACPC-Val-isoAsp(OtBu)-Gly-) (19)

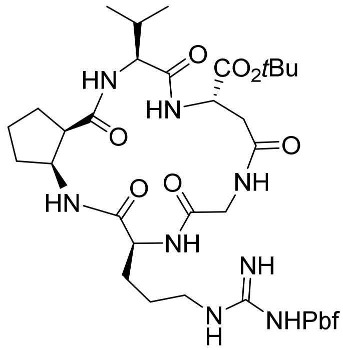

19

Compound **18** (114 mg, 0.13 mmol, considered 1.0 equiv. for stoichiometric calculations) was treated with HATU (154 mg, 0.41 mmol, 3.1 equiv.) and DIPEA (136 μL, 0.78 mmol, 6.0 equiv.) under the conditions described in **GP8**. The product **19** was obtained as pale yellow solid (70 mg, 0.08 mmol, 0.1 equiv.) and it was used without purification in the following step.

Cyclo-(-Arg-(1R,2S)- -ACPC-Val-isoAsp-Gly-) (10)

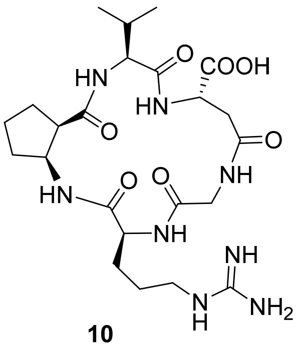

10

Compound **19** (70 mg, crude) was deprotected under the conditions described in **GP9**. The crude product was purified by HPLC [gradient: 100% H_2O + 0.1% CF_3COOH/0% CH_3CN + 0.1% CF_3COOH to 50% H_2O + 0.1% CF_3COOH/50% CH_3CN + 0.1% CF_3COOH in 11 min; flow: 15 mL/min]. Pure fractions were concentrated and freeze-dried, affording **10** as a white solid (9.97 mg, 0.015 mmol, 16% overall yield).

Compound 10:

^{1}H-NMR (500 MHz, H_2O/D_2O) 8.44 (m, 1H), 8.42 (t, 1H), 8.04 (t, 1H), 7.92 (m, 1H), 7.74 (d, 1H), 7.14 (m, 4H), 4.74 (m, 1H), 4.22 (m, 1H), 4.19 (m, 1H), 4.13-3.98 (m, 2H), 3.75 (m, 1H), 3.15 (q, 2H), 2.87 (m, 1H), 2.85-2.73 (m, 2H), 1.94 (m, 1H), 1.93-1.82 (m, 2H), 1.88 (m, 2H), 1.72-1.67 (m, 2H), 1.72-1.63 (m, 2H), 1.66-1.58 (m, 2H), 0.96-0.91 (dd, 6H). ^{13}C-NMR (125 MHz, D_2O) 60.7, 53.8, 53.5, 50.2, 46.9, 42.7, 40.2, 37.7, 29.4, 27.5, 27.3, 27.1, 24.7, 21.9, 18.9; MS (ESI) m/z calcd. for $[C_{23}H_{39}N_8O_7]^+$: 539.2936 $[M + H]^+$; found: 539.2543 $[M + H]^+$.

Figure S3. HPLC trace of compound 10:.

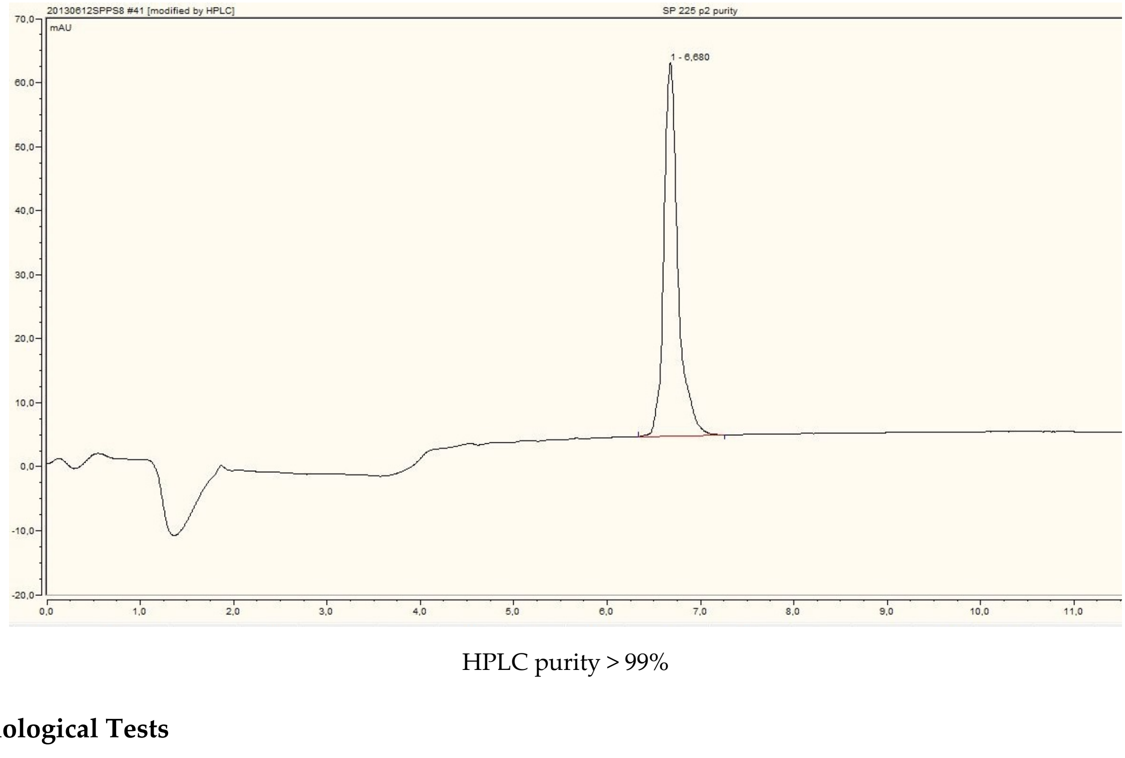

HPLC purity > 99%

Biological Tests

Cell Culture

The skin cancer cells WM-115, model for $\alpha_v\beta_3$ and also expressing $\alpha_v\beta_5$ and $\alpha_5\beta_1$, were prepared following the procedure reported in the literature.[2]

[2] Conradi, J., Huber, S., Gaus, K., Mertink, F., Gracia, S. R., Strijowski, U., Backert, S., Sewald, N., *Amino Acids.* 2012, *43*, 219–232.

Cell adhesion assays with WM-115 human epithelial cancer cells

For the competition assay, 96-well ELISA-plates were immobilized overnight at 37 °C with recombinant vitronectin in PBS (1 μg/mL), afterwards blocked with 2 % (w/v) BSA in PBS.

WM-115 cells were washed with MEM (Minimum Essential Medium)-buffer, detached with trypsin and resuspended to a cell density of 1 × 10^5 cells/mL. WM-115 cells were then incubated with fluorescein diacetate for 30 min at 37 °C in the dark, washed with MEM medium and resuspended with MEM medium containing $CaCl_2$ and $MgCl_2$ (2 mM) and incubated on ice for 30 min in the dark.

The cell suspension was added to the peptide solutions to give concentrations from 0.5 mM to nanomolar (dilution row [mM]: 0.5, 0.25, 0.125, 0.0625, 0.03125, 0.015625, 0.0078125, 0.00390625, 0.00195313, 0.00097565, 0.00048828, 0.00024414). Cells and peptide were incubated for 30 min at 37° C in the dark and afterwards added to the 96-well ELISA-plates previously coated with vitronectin and incubated for 1 h at 37 °C in the dark. Unbound cells were aspirated, and bound cells were washed three times with MEM medium and fluorescence measured (λ_{ex}: 485 nm, λ_{em}: 514 nm) with an Infinite™ 200 Microplate Reader (Tecan, Männedorf, Switzerland).

Determination of IC_{50} Values

IC_{50} values (inhibition concentration of 50% cell adhesion) for the tested compounds **8-10** were determined with OriginPro 2016 (sigmoidal fit: DoseResp).

The IC_{50} values were determined four times per peptide in two independent assays here reported, and the final values are the arithmetic mean ± the standard deviation (SD) of the two independent assays. The reference peptide *c*(RGDfV) **1b** was tested together with the peptides.

Peptide	1° Assay M	2° Assay M
SP190 (**8**)	52	98
SP179 (**9**)	117	132
SP225 (**10**)	> 300	> 300
c(RGDfV) (**1b**)	4.0	7.1

Figure S4. 1st Assay.

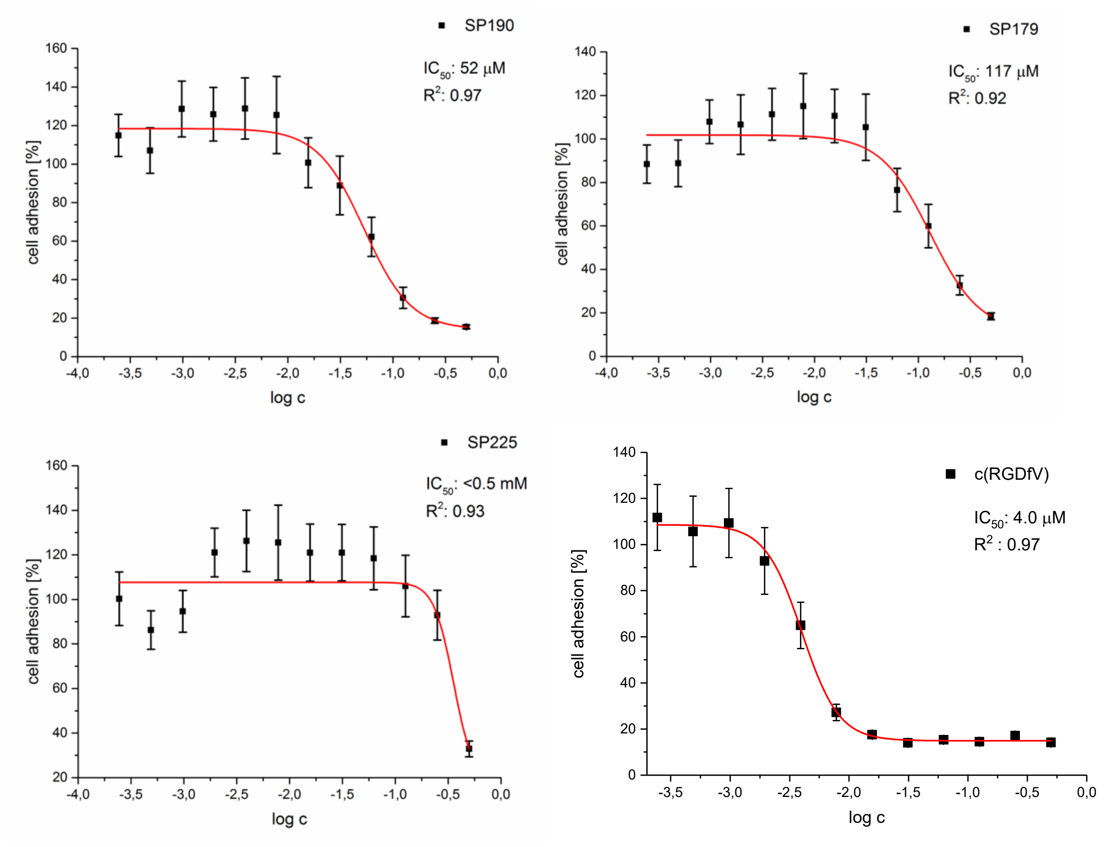

Figure S5. 2nd Assay.

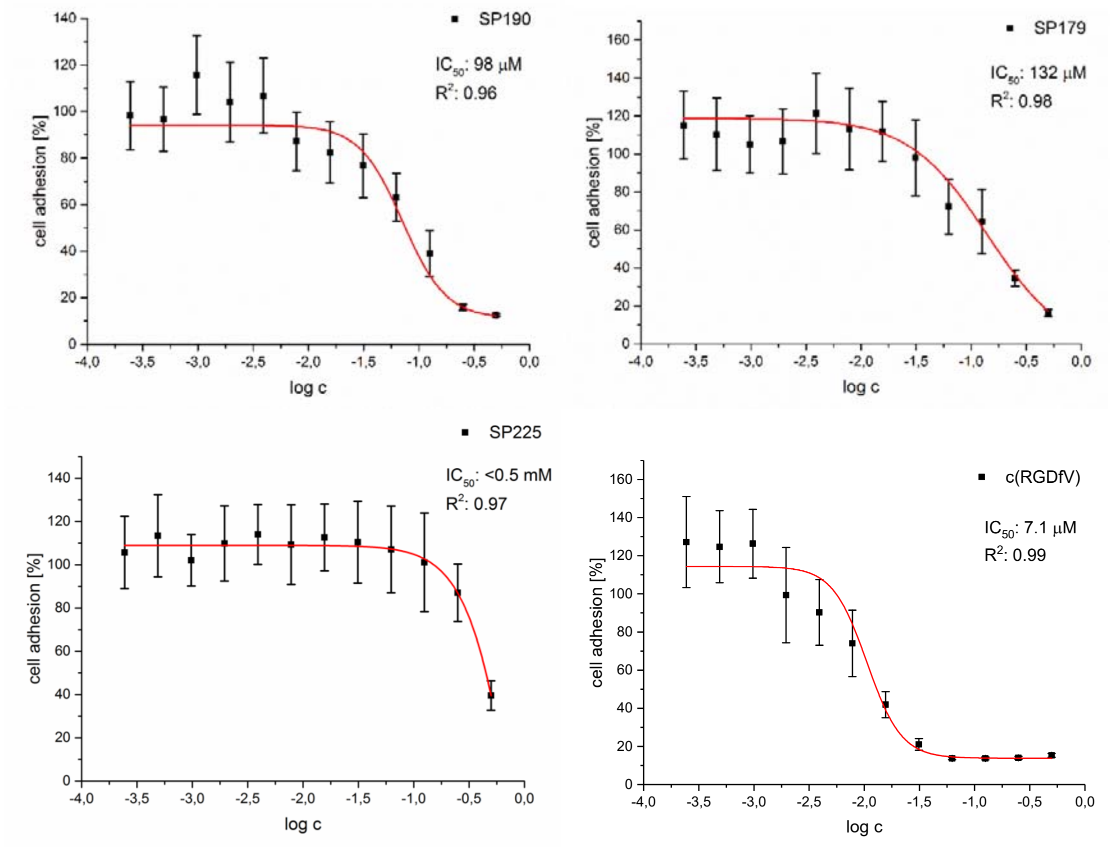

Computational Studies

Docking Calculations

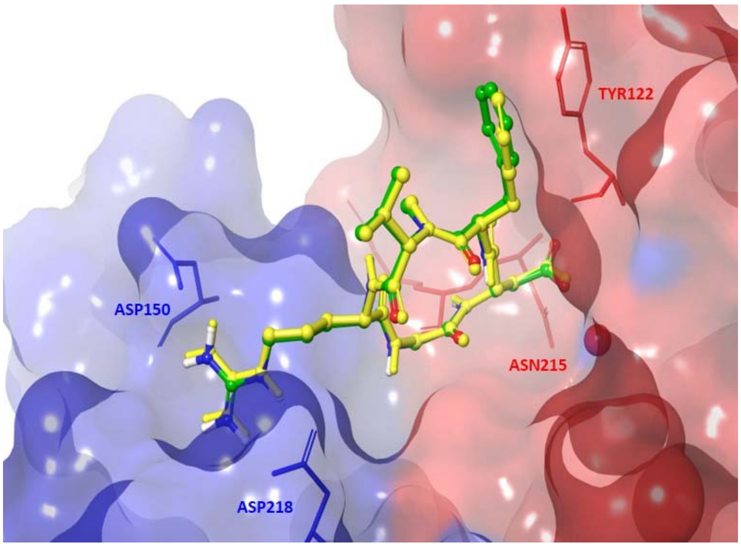

Figure S6. Docking best pose of Cilengitide (green C atoms) in the crystal structure of the extracellular domain of αvβ3 integrin (α unit blue, β unit red) overlaid on the bound conformation of Cilengitide (yellow). The RMSD calculated on heavy atoms is 0.3287 Å. Only selected integrin residues involved in interactions with the ligand are shown. The metal ion at MIDAS is shown as a red CPK sphere.

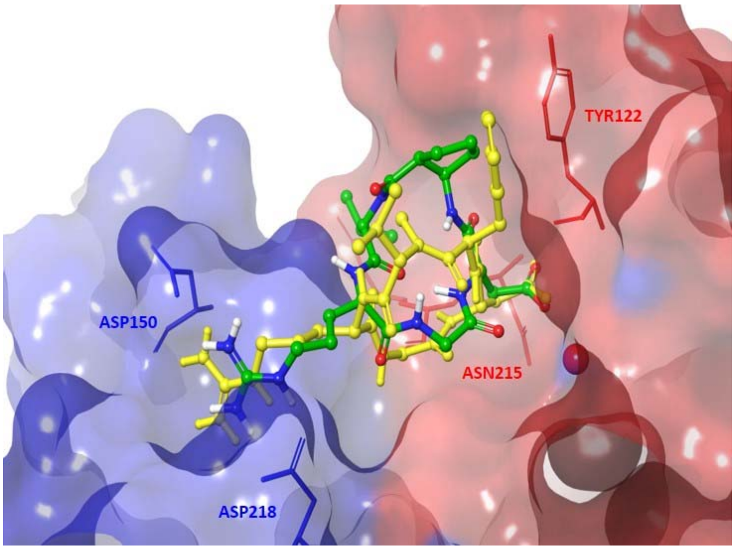

Figure S7. Docking best pose of compound **8** (Type II, green C atoms, Gscore = -8.48 kcal/mol) in the crystal structure of the extracellular domain of $\alpha_v\beta_3$ integrin (α unit blue, β unit red) overlaid on the bound conformation of Cilengitide (yellow). Only selected integrin residues involved in interactions with the ligand are shown. The metal ion at MIDAS is shown as a red CPK sphere.

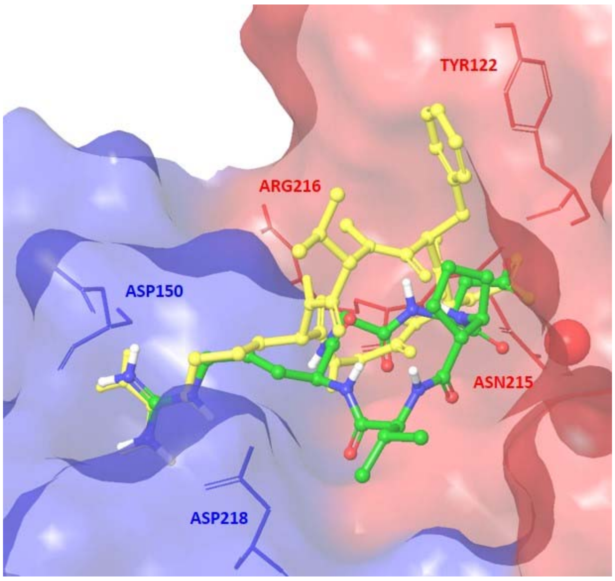

Figure S8. Docking best pose of compound **9** (Type I, green C atoms, Gscore = -9.86 kcal/mol) in the crystal structure of the extracellular domain of $\alpha_v\beta_3$ integrin (α unit blue, β unit red) overlaid on the bound conformation of Cilengitide (yellow). Only selected integrin residues involved in interactions with the ligand are shown. The metal ion at MIDAS is shown as a red CPK sphere.

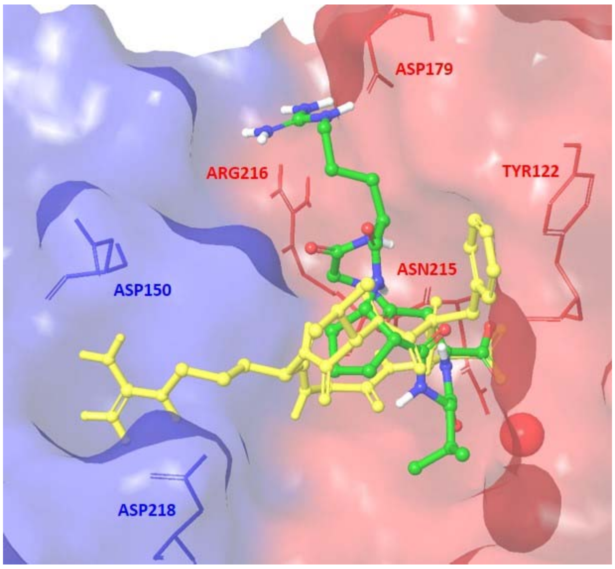

Figure S9. Docking best pose of compound **10** (Type I′, green C atoms, Gscore = -5.74 kcal/mol) in the crystal structure of the extracellular domain of $\alpha_v\beta_3$ integrin (α unit blue, β unit red) overlaid on the bound conformation of Cilengitide (yellow). Only selected integrin residues involved in interactions with the ligand are shown. The metal ion at MIDAS is shown as a red CPK sphere.
